# Roles of MicroRNAs in Disease Biology

**DOI:** 10.31662/jmaj.2023-0009

**Published:** 2023-03-24

**Authors:** Hiroshi I. Suzuki

**Affiliations:** 1 Division of Molecular Oncology, Center for Neurological Diseases and Cancer, Nagoya University Graduate School of Medicine, Nagoya, Japan; 2 Institute for Glyco-core Research (iGCORE), Nagoya University, Nagoya, Japan; 3 Center for One Medicine Innovative Translational Research, Gifu University Institute for Advanced Study, Gifu, Japan

**Keywords:** microRNA, biogenesis, target regulation, cancer, genetic disease

## Abstract

Gene regulation by microRNAs (miRNAs) plays important roles in development, physiology, and disease. miRNAs are an abundant class of noncoding RNAs that are generated through multistep biosynthetic pathways and typically repress gene expression through target destabilization and translational inhibition. Complex interactions between miRNAs and target mRNAs are associated with characteristic molecular mechanisms, including miRNA cotargeting, target-directed miRNA degradation, and crosstalk with various RNA-binding proteins. Consistent with the broad influence on cellular function, miRNA deregulation is commonly observed in various diseases, particularly cancer, with both tumor-suppressive and oncogenic roles. Mutations in the miRNA biosynthetic pathway and several miRNA genes have been linked to diverse types of cancer and a subset of genetic diseases, respectively. Additionally, super-enhancers play important roles in the regulation of cell type-specific and disease-associated miRNAs. This review summarizes the molecular features of miRNA biogenesis and target regulation along with the roles of miRNAs in disease biology, with recent examples expanding the pathophysiological roles of miRNAs.

## Introduction

MicroRNAs (miRNAs) are an abundant class of noncoding RNAs that are approximately 22-nucleotide long and are generated from miRNA genes through multistep biosynthetic pathways. miRNAs shape complex post-transcriptional regulation networks using the short sequence complementarity between miRNAs and their target mRNAs for target interaction ^(1), (2), (3)^. Such complex interactions between miRNAs and target mRNAs are associated with characteristic molecular mechanisms, including miRNA cotargeting, target-directed miRNA degradation (TDMD), and crosstalk with various RNA-binding proteins (RBPs), leading to various regulatory outcomes beyond the typical outcome of miRNA targeting, i.e., simple and modest target repression ^(4)^. Numerous studies have shown that miRNA deregulation is commonly observed in various diseases, including cancer, in which miRNAs have both tumor-suppressive and oncogenic roles. Mutations in the miRNA biosynthetic pathway and several miRNA genes have been linked to diverse types of cancer and a subset of genetic diseases, respectively. Super-enhancers play important roles in the regulation of cell type-specific and disease-associated miRNAs ^(5), (6)^. Together with recent examples expanding the pathophysiological roles of miRNAs, this review summarizes the molecular features of miRNA biogenesis and target regulation in mammalian cells and the roles of miRNAs in disease biology.

## Molecular Features of the miRNA Biosynthetic Pathway

**Figure 1.**
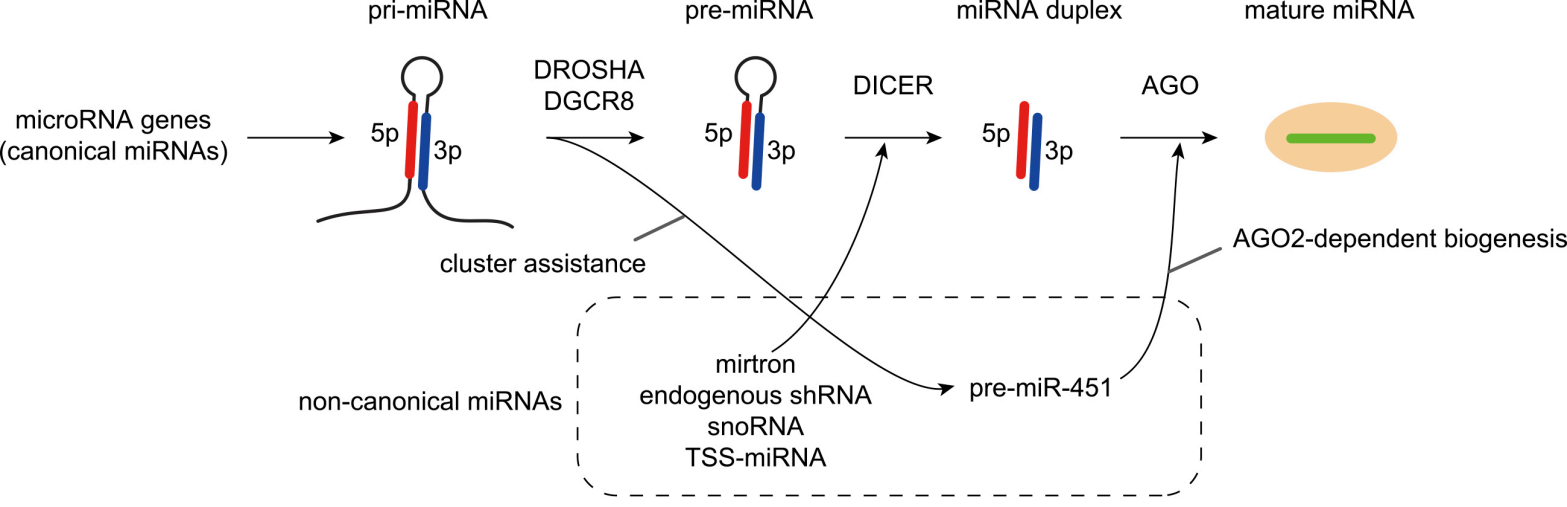
Overview of the miRNA biogenesis pathway in mammalian cells. The canonical miRNA biogenesis pathway includes multiple steps, including the transcription of pri-miRNAs, DROSHA-mediated processing in the nucleus, export to the cytoplasm, DICER-mediated processing in the cytoplasm, loading onto AGO proteins, and RISC formation. Several sources of noncanonical miRNAs that are generated independently of DROSHA or DICER are known. Erythrocyte-specific miR-451 is generated via the cluster assistance mechanism of DROSHA processing and DICER-independent AGO2-mediated processing.

In mammalian cells, the biosynthetic pathway of canonical miRNAs begins with transcription by RNA polymerase II of hairpin-embedded RNAs called primary miRNAs (pri-miRNAs), which are processed by the DROSHA/DGCR8 complex into a hairpin precursor miRNA (pre-miRNA) (Figure 1) ^(1), (2), (3)^. The pre-miRNAs are exported to the cytoplasm via Exportin-5 and RAN-GTP and further processed by DICER. DICER yields the miRNA duplex, which has a 2-nucleotide 3′-overhang at each end. After loading the miRNA duplex into Argonaute (AGO) proteins (AGO1-4 in mammals), one strand of the miRNA duplex is retained as miRNA (the guide strand) to form the RNA-induced silencing complex (RISC). The other strand, termed the miRNA* or passenger strand, is expelled from the AGO proteins. Strand choice is determined by the preferred orientation of whether either of the 5′-ends of the miRNA duplex is suitable for binding to the MID domain of AGO proteins. AGO proteins prefer strands with 5′-uridine or 5′-adenosine and thermodynamically unstable 5′-ends, thereby directly contributing to small RNA asymmetry ^(7), (8), (9)^. In addition to the biogenesis of canonical miRNAs, several classes of noncanonical miRNAs, such as mirtrons, erythrocyte-specific miR-451, and transcription start site miRNAs (TSS-miRNAs), are generated independently of DROSHA or DICER (Figure 1) ^(1), (10)^.

On the basis of high-throughput sequencing of small RNAs, the current version (v22.1) of miRBase lists more than 1,900 and 1,200 miRNA gene annotations in humans and mice, respectively ^(11)^. Among them, 500 (to approximately 1,000) miRNA genes meet the stringent criteria for structure, including consistent 5′-terminus and 2-nt overhang of miRNA duplex, levels of expression, and conservation of sequences ^(2)^. Recent advances in our understanding of miRNA biosynthesis have demonstrated that several sequence features of pri-miRNAs are important for efficient pri-miRNA processing, supporting the authenticity of confidently annotated (canonical) miRNAs ^(1), (2)^. Such sequence features include a narrow range of pri-miRNA stem lengths (35 ± 1 base pairs), a CNNC motif downstream of the DROSHA processing site, a UG motif at the base of the pri-miRNA hairpin, a mismatched GHG motif in the basal stem region, and a UGU(GUG) motif in the apical loop. Supporting the significance of such sequence features, among 1,881 human pri-miRNAs in miRBase v21, only 758 pri-miRNAs are productively processed by DROSHA using stringent criteria ^(12)^. Conversely, an additional mechanism, termed “cluster assistance” mechanism, contributes to efficient DROSHA-mediated processing of suboptimal pri-miRNA hairpins, which are typically encoded together with optimal pri-miRNA hairpins in polycistronic pri-miRNAs, in cells ^(13), (14), (15), (16), (17)^. In this case, optimal pri-miRNA hairpins can assist in the DROSHA-mediated processing of neighboring suboptimal pri-miRNA hairpins, relaxing the stringent requirement of pri-miRNA sequence features for DROSHA processing ^(13), (14), (15), (16), (17)^ . Two mediators of proximity-based enhancers of DROSHA processing, SAFB2 and ERH, have been identified ^(15), (16), (17)^. This “cluster assistance” mechanism may help to explain that miRNA cluster genes (polycistronic miRNA genes) are prevalent across evolution.

## miRNA Activity and Target Regulation

RISC binds mainly to the 3′-untranslated region (3′-UTR) of target mRNAs and typically represses them together with TNRC6 (GW182) proteins through target destabilization and translational inhibition ^(1), (2), (3)^. Because target recognition is usually based on the seed sequence of miRNAs (nucleotides 2-7), one miRNA can target hundreds of target mRNAs. Therefore, miRNAs have broad influences on diverse cellular functions. Recent reviews have provided a compilation of mouse phenotypes upon deletion of one or more miRNA genes, which include lethality; abnormal development; alterations in various physiological processes such as lipid metabolism; and differential responses to disease models, including cancer, infection, and tissue injuries ^(2), (18)^. In postembryonic cells, target destabilization is dominant and depends on the abundance of miRNAs; detectable miRNA-mediated target destabilization requires high miRNA expression levels ^(2), (19)^. Consistent with the requirement of high miRNA expression for target repression, a small subset of cell type-specific miRNAs dominates the miRNA expression pool and post-transcriptional regulation by AGO proteins, as demonstrated by miRNA expression profiling and functional profiling using miRNA sensor libraries and crosslinking and immunoprecipitation (CLIP) experiments ^(20), (21), (22)^. Super-enhancers, an important enhancer subclass that controls cell identity and disease genes, play central roles in tissue-specific and evolutionarily conserved patterns of miRNA expression and function ^(5)^. Super-enhancer-associated miRNAs (SE-miRNAs) include many important cell-specific miRNAs, whose knockout perturbs the function of the respective cell type and tissue ^(5)^.

**Figure 2.**
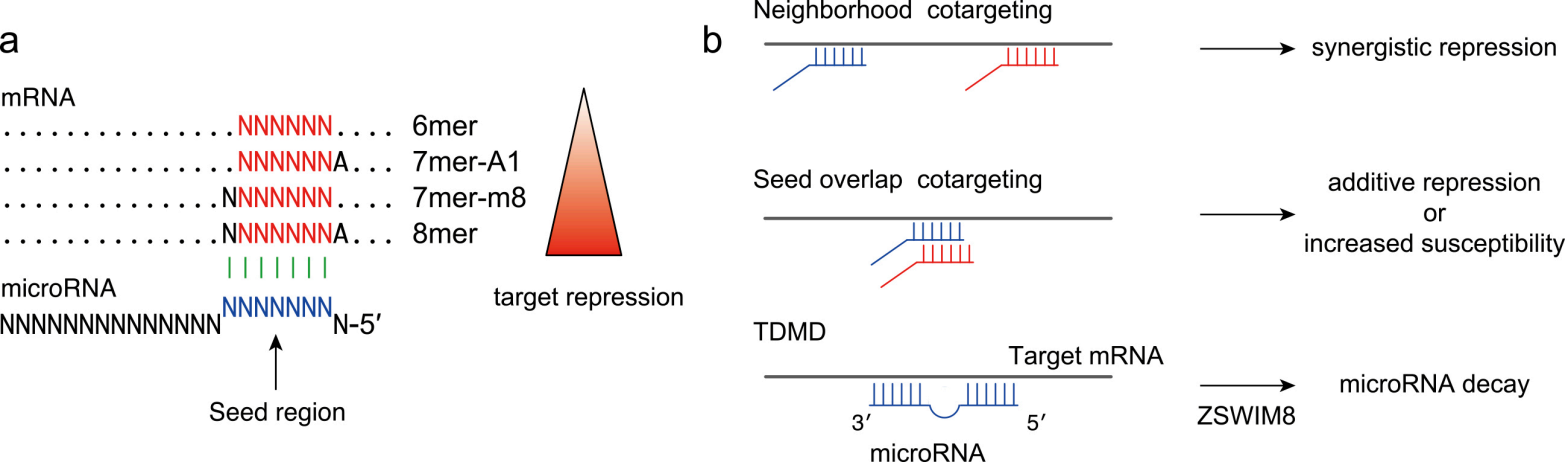
Effects of miRNA-mRNA interactions on target regulation and miRNA stability. a, Classification of canonical miRNA target sites. b, Effects of multiple miRNA target sites and extensive miRNA-mRNA target interactions on target repression and miRNA stability.

The degree of seed-based target repression depends on the target type, where either an additional match to miRNA nucleotide 8 or an A across miRNA nucleotide 1 augments the 6-nucleotide seed match (Figure 2a) ^(2)^. Because target recognition is mediated via a very short sequence as well as transcription factor-DNA interaction and target repression via a single site is typically modest, additional pairing to the 3′ region of the miRNA and crosstalk among multiple target sites, multiple miRNAs, and RBPs have complex regulatory influences on miRNA activities, including miRNA cotargeting, TDMD, and crosstalk with various RBPs (Figure 2b) ^(4)^.

First, target interactions via two miRNA binding sites can mediate synergistic inhibition when the two sites are closely spaced at a distance of approximately 15-100 nt between seed starts. Multivalent interactions between GW182 and multiple AGO proteins underlie this synergistic effect: AGO2-GW182 interactions have also been reported to promote the phase separation of RISC ^(23)^. This type of miRNA cotargeting (“neighborhood” miRNA cotargeting) appears to be evolutionarily maintained and plays an important role in the brain ^(24)^. An additional scenario of miRNA cotargeting is “seed overlap” miRNA cotargeting, in which extensively overlapping seed sites can increase susceptibility to two miRNAs ^(25)^. A recent systematic characterization revealed that extensive “seed overlap” is a prevalent feature of broadly conserved miRNAs ^(25)^. This study also demonstrated that highly conserved target sites of broadly conserved miRNAs are largely divided into two classes―those conserved among eutherian mammals and those from human to *Coelacanth*―and that the latter has a stronger association with both “seed overlap” and “neighborhood” miRNA cotargeting ^(25)^.

Second, recent studies have highlighted that in contrast to the typical consequences of miRNA-target interactions, target RNAs can inversely facilitate the decay of miRNAs (TDMD) ^(26)^. TDMD is triggered by highly complementary target RNAs and is frequently associated with the 3′-end addition of nontemplated uridines or adenosines (tailing) and 3′-to-5′ exonucleolytic shortening (trimming) of miRNAs. ZSWIM8 Cullin-RING E3 ubiquitin ligase has recently been identified as a direct mediator of TDMD ^(27), (28)^. Functional analyses have suggested that ZSWIM8 recognizes the conformational changes in AGO proteins, which are induced by TDMD triggers, and directs the polyubiquitination of AGO proteins, leading to the destruction of both AGO proteins and miRNAs ^(27), (28)^. TDMD appears to play an important role in the destabilization of many short-lived miRNAs ^(27)^. Crosstalk with RBPs is discussed later.

## miRNAs in Cancer Biology

**Figure 3.**
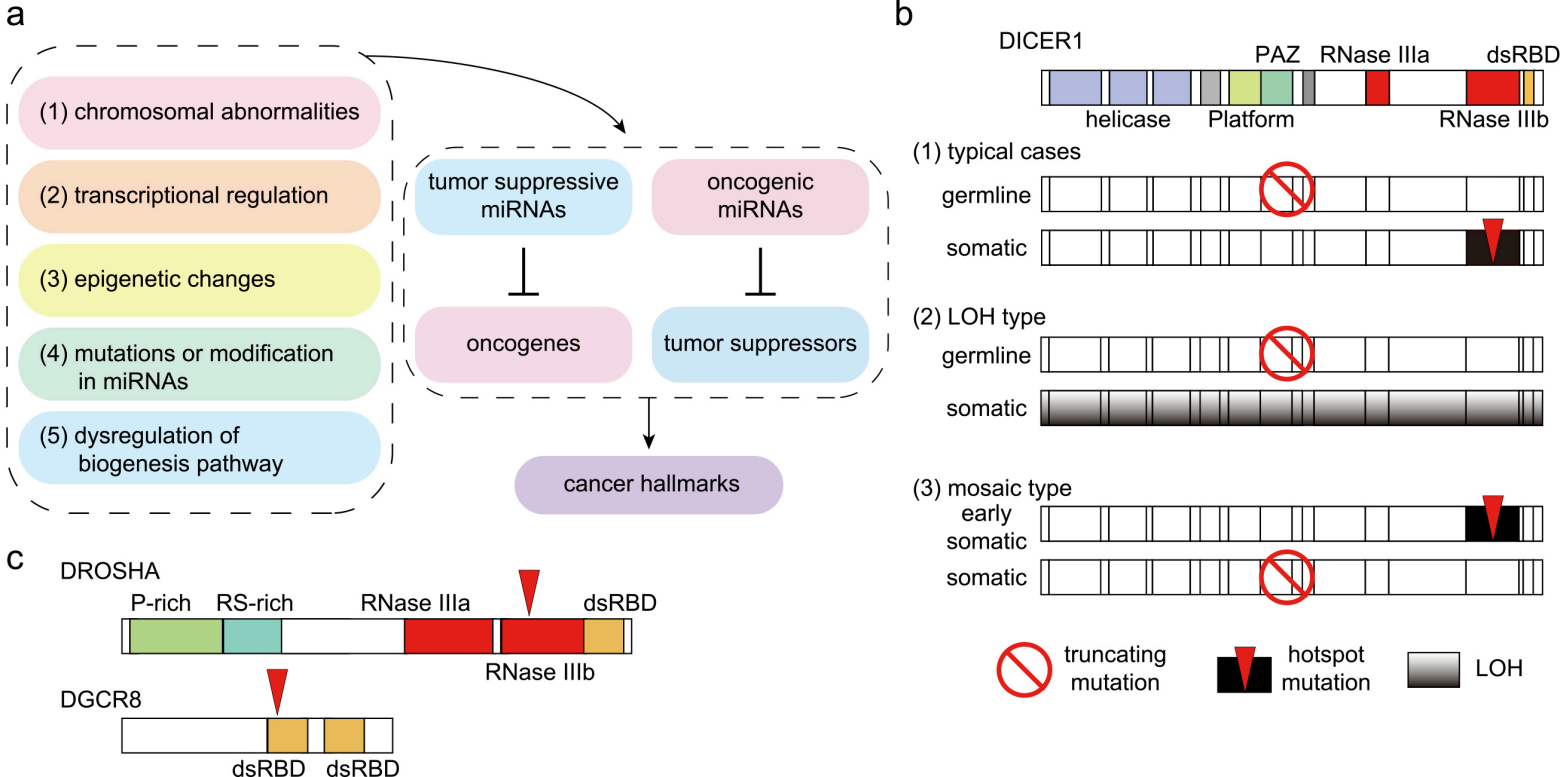
Mechanisms of miRNA dysregulation in cancer. a, The altered expression of tumor-suppressive or oncogenic miRNAs is induced by various mechanisms: (1) chromosomal abnormalities; (2) alterations in transcriptional regulation; (3) epigenetic mechanisms including super-enhancers; (4) mutations or modifications in miRNAs; and (5) dysregulation of the miRNA biogenesis pathway. b, Dysregulation of DICER in *DICER1* syndrome. Several models of two-hit activation are shown. Various other scenarios have also been reported. c, Mutations in *DROSHA* and *DGCR8* in Wilms tumors.

miRNAs serve as tumor suppressors or oncogenes by regulating oncogenes and tumor suppressors as their targets, respectively, as deregulation of miRNA expression and function is commonly observed in cancer (Figure 3a) ^(29)^. Tumor-suppressive and oncogenic miRNAs have been shown to affect many aspects of the hallmark traits of cancer. Among these hallmarks, miRNAs regulate the autonomous behavior of cancer cells, including “evading growth suppressors,” “sustaining proliferative signaling,” “resisting cell death,” “enabling replicative immortality,” and “activating invasion and metastasis” ^(29), (30)^. Additionally, miRNAs in cancer cells have non-cell-autonomous functions by modulating the vasculature, extracellular matrix, and immune cells in the tumor milleu ^(31), (32)^. Such remodeling of tumor microenvironments by miRNAs contributes to other hallmark traits related to the tumor milleu, such as “avoiding immune destruction,” “tumor-promoting inflammation,” “inducing or accessing vasculature,” and “activating invasion and metastasis.”

The altered expression of miRNAs in cancer is induced by various mechanisms (Figure 3a): (1) amplification or deletion of miRNA genes; (2) alterations in transcriptional regulation; (3) epigenetic mechanisms including super-enhancers; (4) mutations or modifications in miRNAs; and (5) dysregulation of the miRNA biogenesis pathway. In cancer, mutations in miRNA genes are relatively rare, except for a few examples such as mutations in miR-142 in hematological malignancies ^(33), (34), (35)^. Cancer-associated mutations in the miRNA biogenesis pathway are described in the following section.

Well-known examples of the first scenario include chromosomal abnormalities, including loss of the miR-15a/16-1 cluster in chronic lymphocytic leukemia (CLL), amplification of the miR-17~92 cluster in malignant lymphoma, and amplification and enhancer hijacking mechanisms of the Chr19q13.41 miRNA cluster (C19MC) in embryonal tumors with multilayered rosettes (EMTRs) ^(36), (37), (38), (39), (40)^. A recent study has shown that a subset of chromosome 21 miRNAs, miR-99a, miR-125b-2, and miR-155, contributes to a trisomy 21-like hematopoietic state and preleukemia development in Down syndrome (trisomy 21), in which children frequently exhibit preleukemic transient abnormal myelopoiesis and develop myeloid leukemia ^(41)^. In the second scenario, many studies have identified upstream transcription factors controlling miRNA genes and revealed that many oncogenic and tumor-suppressive transcription factors, including MYC, p53, and epithelial-mesenchymal transition (EMT) transcription factors, regulate both protein-coding and miRNA genes ^(42)^. The third scenario, epigenetic changes, is also frequently observed in cancer and includes aberrant DNA methylation and histone modulation of miRNA genes. A previous systematic analysis of SE‐miRNAs in various cancer cell lines highlighted that super-enhancer gain and loss are frequently observed for oncogenic and tumor-suppressive miRNAs, respectively ^(5)^. The target genes of SE-miRNAs with such super-enhancer alterations are also associated with the cancer hallmark traits ^(5)^. Importantly, miRNAs with super-enhancer gain tend to be associated with worse prognosis in various cancer types, such as pancreatic, colon, and breast cancers ^(5)^. Such SE-miRNAs are also important for distinguishing cancer subtypes ^(43), (44)^.

## Mutations in the miRNA Biosynthetic Pathway in Cancer

Most vertebrates encode only a single gene for DROSHA- and DICER-type proteins; thus, mutations or deletions of DROSHA and DICER substantially affect most canonical miRNAs. In addition to the early reports of miRNA profiling in cancer, mouse models have demonstrated that monoallelic *Dicer1* loss facilitates Kras-driven lung tumor formation and tumor formation in a retinoblastoma-sensitized background, whereas biallelic *Dicer1* loss drives angiosarcoma, suggesting a tumor-suppressive role of *Dicer1* ^(45), (46), (47)^. In human cancers, mutations in *DROSHA*, *DGCR8*, and *DICER1* are associated with several types of cancer. Representative association includes mutations in (1) *DICER1* in a broad spectrum of hereditary cancer predisposition syndrome (so-called *DICER1* syndrome; Figure 3b) and (2) *DROSHA* and *DGCR8* in Wilms tumors (Figure 3c).

The association of germline *DICER1* mutations with familial pleuropulmonary blastoma (PPB) was initially described in 2009 ^(48)^. Subsequent studies have shown that germline *DICER1* mutations are associated with various malignant and benign tumors, including PPB, cystic nephroma, ovarian sex cord-stromal tumor (OSCST; especially Sertoli-Leydig cell tumor (SLCT)), embryonal rhabdomyosarcoma (ERMS), multinodular goiter, differentiated thyroid carcinoma, nasal chondromesenchymal hamartoma, ciliary body medulloepithelioma, and others ^(49), (50), (51)^. Many *DICER1*-related tumors, including PPB and cystic nephroma, develop in early childhood, whereas OSCST, ERMS, and multinodular goiter have late onset ^(49), (50)^. In typical cases, one germline allele of *DICER1* is not functional because nonsense or frameshift mutations occur across the entire genes as the first hit (Figure 3b) ^(49), (50), (52)^. The other allele acquires somatic hotspot mutations, which occur at the metal-ion-binding residues D1709 and E1813 or adjacent residues in the RNase IIIb domain ^(49), (50), (51), (52)^. The DICER protein has two RNase III domains, RNase IIIa and IIIb, which are responsible for yielding the 3p-arm and 5p-arm miRNAs, respectively. From a functional standpoint, the RNase IIIb domain mutation in *DICER1* syndrome reduces the production of 5p-arm miRNAs, including the important tumor-suppressive miRNA let-7 family, whereas 3p-arm miRNAs are only partially reduced ^(51), (52), (53)^. Although infrequent in overall *DICER1* syndrome, loss-of-heterozygosity occurs as the second hit in pineoblastoma (Figure 3b) ^(54)^. In pineoblastoma, the recurrent homozygous deletion of *DROSHA* has also been reported ^(55)^. In some patients, instead of germline mutations, mosaic mutations increase predisposition to cancer (Figure 3b) ^(56), (57)^. Additionally, *DICER1* mutations are observed in Wilms tumors as described in the next paragraph. Collectively, *DICER1* syndrome represents a unique two-hit model of cancer predisposition syndrome.

Mutations in *DROSHA* and *DGCR8* are detected in approximately 20% of Wilms tumors with blastemal histology ^(58), (59), (60), (61), (62)^. The mutations are concentrated in the residues in the RNase IIIb domain of DROSHA (E1147) and in the double-stranded RNA-binding domain (dsRBD) of DGCR8 (E518) (Figure 3c) ^(58), (59), (61), (62)^. DICER RNase IIIb domain mutants affect 5p-arm processing, whereas DROSHA RNase IIIb mutants have defects in both arm processing and function, possibly in a dominant-negative manner ^(59), (61), (62)^. Possibly reflecting a regulatory network comprising let-7, IGF2BPs, and MYC, alterations in *IGF2* and *MYCN* are frequent in blastemal type tumors ^(59)^. Additionally, alterations in other miRNA biogenesis pathways, such as *DICER1*, *XPO5*, and *TARBP2*, have been observed in Wilms tumors ^(59), (60), (61)^.

Although *DROSHA*/*DGCR8*/*DICER1* mutations appear to be infrequent in adult tumors, a recent pan-cancer analysis using The Cancer Genome Atlas and MSK-IMPACT databases reported enrichment of hotspot mutations in *DICER1* RNase IIIb and RNase IIIa domains in uterine cancers, including endometrial cancer and uterine sarcoma ^(63), (64)^. Some cases exhibit biallelic mutations with truncation or nonsense mutations. The hotspot mutation of the RNase IIIa domain, S1344L, depletes 5p-arm miRNAs as well as RNase IIIb hotspot mutation and derepresses target genes of 5p miRNAs (let-7 and other miRNAs), such as *HMGA2* and *IGF2BP2* ^(63)^. Such effects of the RNase IIIa domain mutation can be partly explained by the fact that this site is structurally very close to the RNase IIIb catalytic site ^(63)^.

## Mutations in miRNA Genes in Genetic Diseases

In several genetic disorders, associated chromosomal abnormalities, including large deletions, affect some miRNA gene loci, as summarized in other reviews ^(35), (65), (66)^. Although the pathological roles of these miRNAs are largely unclear, the importance of alterations in the miR-17~92 cluster has been demonstrated in Feingold syndrome, whose core features are microcephaly, short stature, and digital abnormalities ^(67)^. Although germline loss-of-function mutations of *MYCN* are detected in approximately 70% of patients with Feingold syndrome, germline hemizygous 13q31.3 microdeletions including the miR-17~92 cluster have been identified in the remaining cases. Importantly, the targeted deletion of the miR-17~92 cluster phenocopied widespread skeletal defects associated with human phenotypes ^(67)^.

Other mutation-based alterations in miRNA activities include mutations in miRNA genes and miRNA target sites ^(65), (66)^. Because miRNA genes are small mutational targets, mutations in a few miRNA genes have been linked to human genetic diseases ^(35), (65), (66)^. Point mutations in the seed region of miR-96, which is expressed in hair cells of the inner ear, have been identified in autosomal dominant progressive hearing loss, DFNA50 ^(68), (69)^. The identified mutations impair the production of mature miRNA-96 ^(68), (69)^. The significance of miR-96 mutations is supported by the identification of a point mutation in miR-96 in a mouse mutant called diminuendo, with progressive loss of hearing and hair cell anomalies ^(70)^. Additionally, mutations in miR-184 have been associated with endothelial dystrophy, iris hypoplasia, congenital cataract, and stromal thinning (EDICT) syndrome and familial and sporadic cases of keratoconus ^(71), (72), (73)^. Mutations in miR-96 and miR-184 occur at multiple nucleotides of single miRNAs and are predicted to primarily cause loss-of-function.

**Figure 4.**
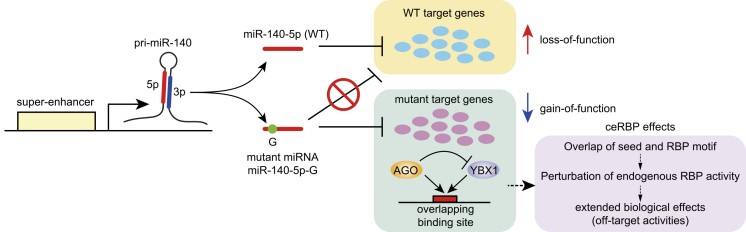
Pathogenic mutation of miR-140 in skeletal dysplasia and ceRBP effects. (Cited and modified from Grigelioniene G, Suzuki HI, Taylan F, et al. Gain-of-function mutation of microRNA-140 in human skeletal dysplasia. Nat Med. 2019;25(4):583-90^(75)^ and Suzuki HI, Spengler RM, Grigelioniene G, et al. Deconvolution of seed and RNA-binding protein crosstalk in RNAi-based functional genomics. Nat Genet. 2018;50(5):657-61^(76)^. Copyright of the figure belongs to the authors.) A seed region mutation in chondrocyte-specific miR-140 has been identified in spondyloepiphyseal dysplasia *MIR140* type Nishimura (OMIM #618618). This mutation results in widespread derepression of wild-type miR-140-5p targets and repression of mutant miR-140-5p (miR-140-5p-G) targets. Additionally, the miR-140-5p mutant seed competes with the Ybx1 RNA-binding protein for the overlapping binding sites.

Certain miRNA mutations have been proposed to exert gain-of-function effects in human diseases. In inherited retinal dystrophy associated with ocular coloboma, a seed region mutation in miR-204 is suggested to be associated with gain-of-function effects ^(74)^. A well-confirmed example of gain-of-function mutation is a recently identified seed region mutation in miR-140 in a novel form of spondyloepiphyseal dysplasia called spondyloepiphyseal dysplasia (SED) *MIR140* type Nishimura (OMIM #618618) (Figure 4) ^(75)^. Genome sequencing revealed a common substitution in the first nucleotide of the seed region of miR-140-5p in two unrelated families. The skeletal dysplasia is characterized by disproportionate short stature with short limbs, small hands and feet, and midface hypoplasia with a small nose, together with several radiological hallmarks including mild spondylar dysplasia, delayed epiphyseal ossification of the hip and knee, and severe brachydactyly with cone-shaped phalangeal epiphyses. miR-140 is driven by a chondrocyte-specific super-enhancer in mice and humans and is highly abundant in chondrocytes, explaining the widespread skeletal abnormalities. The mutant miR-140 gene yields abundant mutant miR-140-5p expression without miRNA processing defects. Importantly, comparative studies of miR-140 knockout and mutant mice have revealed distinct skeletal phenotypes, whose abnormalities in mutant mice with the same mutation are similar to those of patients ^(75)^. Consistent with the additional skeletal abnormalities in miR-140 mutant mice, the mutation resulted in widespread derepression of wild-type miR-140-5p targets and repression of mutant miR-140-5p targets in the chondrocyte transcriptome. Collectively, these findings indicate the loss-of-function and gain-of-function (neomorphic) effects of the miR-140 mutation (Figure 4). Further functional analysis of the mutant miRNA has demonstrated that the new seed sequence of mutant miR-140-5p overlaps with the binding site of Ybx1 RBP and that mutant miRNAs compete with Ybx1 RBPs for overlapping target sites ^(75)^. An additional study has shown that such seed sequence-dependent functional crosstalk between miRNAs and RBPs (crosstalk with endogenous RBPs; ceRBP) is widespread and contributes to seed-dependent off-target activities (Figure 4) ^(76)^.

## Expanding the Pathophysiological Roles of miRNAs

As described earlier, knockout of many conserved miRNAs has been reported to cause not only abnormal developmental phenotypes but also altered responses in various disease models ^(2), (18)^. Recent studies have expanded the pathophysiological roles of miRNAs in many aspects of biology, including disease pathogenesis and human evolution. Finally, this section summarizes recent studies demonstrating the involvement of miRNAs in immune regulation, cardiac remodeling, and metabolic disorders.

Sepsis is a life-threatening organ dysfunction caused by dysregulated host response to infection. Some patients with sepsis develop sepsis-associated immunosuppression, which resembles the phenomenon of lipopolysaccharide (LPS) tolerance. LPS tolerance is an immunosuppressive form of innate immune memory that can be modeled *in vitro* by prolonged LPS treatment. Seeley et al. demonstrated that upregulation of miR-221 and miR-222 in macrophages during prolonged treatment with LPS causes the transcriptional silencing of a subset of inflammatory genes by targeting the chromatin remodeling factor SMARCA4, leading to LPS tolerance ^(77)^. As sepsis-associated immunosuppression is associated with reduced inflammatory cytokine output and increased risk of secondary infection, organ failure, and mortality, increased expression of miR-221 and miR-222 in patients with sepsis correlates with immunosuppression and increased organ damage ^(77)^. miR-221 and miR-222 appear to serve as potential biomarkers for identifying patients with sepsis who have immunosuppression and poor prognosis.

miRNAs can serve as therapeutic targets in addition to biomarkers. Following functional screening of miRNAs inducing cardiac regeneration, Gabisonia et al. demonstrated that overexpression of miR-199a in infarcted pig hearts could stimulate cardiac repair ^(78)^. Importantly, subsequent persistent and uncontrolled expression of the miRNA resulted in sudden arrhythmic death in most of the treated pigs, which was associated with myocardial infiltration of proliferating cells displaying a poorly differentiated myoblastic phenotype ^(78)^. The study by Gabisonia et al. highlights the importance of dosage control in gene and miRNA therapies, even if the therapy is ostensibly beneficial. Additionally, another group reported the importance of miRNA modification in cardiac hypertrophy ^(79)^. Seok et al. reported that redox-dependent cardiac hypertrophy induces miRNA oxidation. 8-Oxoguanine (o^8^G) modification predominantly occurs at position 7 of miR-1 and redirects its target repertoire via o^8^G-A base pairing ^(79)^. Importantly, the introduction of o^8^G or U-substituted miR-1 causes cardiac hypertrophy in mice, and the inhibition of o^8^G-miR-1 attenuates cardiac hypertrophy phenotypes.

As an additional example, Wang et al. recently reported that miR-128-1, located at the positively selected 2q21.3 locus linked to ancient adaptation to milk consumption, serves as a crucial metabolic regulator in mammals ^(80)^. In mice, the inhibition of miR-128-1 prevents diet-induced obesity, liver steatosis, and inflammation and improves glucose homeostasis. Conversely, overexpression of miR-128-1 prevents primary human adipocyte differentiation and reduces the expression of adipocyte hallmark genes. Therefore, miR-128-1 is proposed to be a regulator that connects two 2q21.3-associated phenotypes: ancient adaptation to milk consumption to survive famine and metabolic diseases ^(80)^. Future studies should expand on the role of miRNAs in normal physiology and pathology.

## Conclusions

This review summarizes the molecular features of miRNA biogenesis and target regulation in mammalian cells and the roles of miRNAs in disease biology. Transcription factors and miRNAs constitute two large sets of regulatory factors in a complex gene regulatory network, one interacting with DNA in the nucleus and the other interacting with RNA in the cytoplasm. Importantly, both transcription factors and miRNAs are closely associated with super-enhancer regulation. Although the existing evidence has highlighted their importance in pathophysiology like the tip of the iceberg, future studies on miRNAs are important to understand and describe the regulatory networks at the system level and in an integrative fashion.

## Article Information

### Article Information

This article is based on the study, which received the Medical Research Encouragement Prize of The Japan Medical Association in 2022.

### Conflicts of Interest

None

### Sources of Funding

This work was supported in part by JSPS KAKENHI [19K24694], Mochida Memorial Foundation for Medical and Pharmaceutical Research, Grant for Basic Science Research Projects from the Sumitomo Foundation, Mitsubishi Foundation, Daiichi Sankyo Foundation of Life Science, Uehara Memorial Foundation, and Takeda Science Foundation.

### Acknowledgement

I thank the members of Suzuki laboratories for their discussions. I apologize to the researchers whose reports have not been cited because of space limitations.

### Author Contributions

H.I.S wrote the manuscript.
